# The Effectiveness of Interventions for Non-Communicable Diseases in Humanitarian Crises: A Systematic Review

**DOI:** 10.1371/journal.pone.0138303

**Published:** 2015-09-25

**Authors:** Alexander Ruby, Abigail Knight, Pablo Perel, Karl Blanchet, Bayard Roberts

**Affiliations:** 1 ECOHOST–The Centre for Health and Social Change, London School of Hygiene and Tropical Medicine, London, United Kingdom; 2 Faculty of Public Health and Policy, London School of Hygiene and Tropical Medicine, London, United Kingdom; 3 Centre for Global Non Communicable Diseases, London School of Hygiene and Tropical Medicine, London, United Kingdom; The University of Tokyo, JAPAN

## Abstract

**Background:**

Non-communicable diseases (NCDs) are of increasing concern in low- and middle-income countries (LMICs) affected humanitarian crises. Humanitarian agencies and governments are increasingly challenged with how to effectively tackle NCDs. Reviewing the evidence of interventions for NCDs in humanitarian crises can help guide future policies and research by identifying effective interventions and evidence gaps. The aim of this paper is to systematically review evidence on the effectiveness of interventions targeting NCDs during humanitarian crises in LMICs.

**Methods:**

A systematic review methodology was followed using PRISMA standards. Studies were selected on NCD interventions with civilian populations affected by humanitarian crises in low- and middle-income countries. Five bibliographic databases and a range of grey literature sources were searched. Descriptive analysis was applied and a quality assessment conducted using the Newcastle-Ottawa Quality Assessment Scale for observational studies and the Cochrane Risk of Bias Tool for experimental studies.

**Results:**

The search yielded 4919 references of which 8 studies met inclusion criteria. Seven of the 8 studies were observational, and one study was a non-blinded randomised-controlled trial. Diseases examined included hypertension, heart failure, diabetes mellitus, chronic kidney disease, thalassaemia, and arthritis. Study settings included locations in the Middle East, Eastern Europe, and South Asia. Interventions featuring disease-management protocols and/or cohort monitoring demonstrated the strongest evidence of effectiveness. No studies examined intervention costs. The quality of studies was limited, with a reliance on observational study designs, limited use of control groups, biases associated with missing data and inadequate patient-follow-up, and confounding was poorly addressed.

**Conclusions:**

The review highlights the extremely limited quantity and quality of evidence on this topic. Interventions that incorporate standardisation and facilitate patient follow-up appear beneficial. However, substantially more research is needed, including data on costs.

## Introduction

It is estimated that two-thirds of deaths worldwide are attributable to non-communicable diseases (NCDs), with cardiovascular disease, cancer, diabetes mellitus, and chronic lung disease comprising the largest burden of NCDs.[1] The increasing prevalence of NCDs in low- and middle-income countries (LMICs) has driven the recent increases in the global NCD burden, and importantly the probability of premature death due to NCD is higher in LMICs than in their high-income counterparts.[2] Even in Sub-Saharan Africa—where communicable and vector-borne diseases are still the largest killers—it is estimated that NCDs will become the leading cause of death by 2030.[3]

There are around 50 million persons who have been forcibly displaced from their homes as refugees and internally-displaced persons (IDPs) due to humanitarian crises,[4] defined here as events stemming from armed conflict, natural disasters, or food insecurity that threaten the health and safety of a community. There are also many millions more who remain in areas impacted by humanitarian crises or have recently returned to them after being displaced. While low-income countries continue to suffer the largest burden of humanitarian crises, trends have shown an increase in middle-income countries affected by humanitarian crises, with examples being armed conflicts in Iraq, Libya, Syria, Ukraine, the Balkans, and the Caucasus.[5] These countries have a particularly high burden of NCDs.[6] In addition, humanitarian crises have become more protracted and so health providers are facing pressure to expand beyond the immediate basic primary care traditionally provided by relief agencies and address longer-term health conditions such as NCDs. Moreover, it is known that a number of characteristics related to humanitarian crises such as stress and disrupted access to treatment can exacerbate NCDs.[7]

The rise of NCDs in LMICs and the recent trends in humanitarian crises mean that the burden of NCDs has likely risen among crisis-affected populations. Governments, humanitarian organisations, and international agencies are now increasingly challenged with how to effectively tackle NCDs.[8] While there are best clinical practices on key interventions for treating NCDs in stable settings,[9] there is extremely limited guidance on tackling NCDs in crisis-affected settings. It is unclear what NCD interventions are effective and feasible in such settings, how best to deliver them, and how well interventions are adhering to clinical best practice. As a result, there are increasing calls for a better understanding of NCDs and interventions for NCDs in humanitarian crises.[3, 5, 8] However, no systematic review has been published that examines the evidence on effectiveness of interventions targeting NCDs during humanitarian crises in LMICs. Such a review can help guide future research, policies, and programming by identifying effective interventions as well as evidence gaps.[10] The aim of this paper was to systematically review evidence on the effectiveness of interventions targeting NCDs during humanitarian crises in LMICs. The specific objectives were to: (i) describe the study characteristics; (ii) examine evidence on effectiveness of NCDs in humanitarian crises; and (iii) assess the quality of the evidence on NCD interventions in humanitarian crises. The review forms part of a larger review of evidence on health interventions in humanitarian crises.[11]

## Methods

This systematic review followed the reporting items for systematic reviews as described in the PRISMA statement.[12]

### Eligibility Criteria

The populations of interest were civilians in LMICs affected by humanitarian crises, defined here as events stemming from armed conflicts, natural disasters, or food insecurity that threaten the health and safety of a community. These included populations remaining in areas affected by crises and those forcibly displaced from them as refugees and IDPs. Studies that focused on current or former military populations were excluded. High-income countries were excluded as the vast majority of humanitarian crises occur in LMICs and the resources available to tackle NCDs in LMICs are very different to those in high-income countries. The time periods of humanitarian crises included acute, chronic, and early recovery time periods.

The interventions of interest were health interventions covering health promotion, prevention, treatment, or rehabilitation activities at the individual or population level specifically for outcomes of NCDs.

The outcomes included morbidity/mortality due to NCDs and surrogate outcomes (e.g. blood pressure, blood glucose levels) at the individual or population level. In addition, we also included information on process outcomes (e.g. adherence to clinical treatment) and feasibility of interventions and measurement methods, if the study included data on changes in health outcomes. We did not include mental health outcomes as these have been reviewed elsewhere.[13]

### Information Sources and Search Strategy

The following bibliographic databases were searched: MEDLINE, Embase, Global Health, PsychInfo, and IBSS. The search terms were: (i) disaster-related terms; AND (ii) research study-related terms; AND (iii) geographic terms; AND (iv) NCD terms. A search of the grey literature was also conducted across a range of humanitarian-related databases and standard search databases such as Google. The full search strategy is provided in S1 File. Studies published in any language between January 1980 and June 2014 were included.

### Study Selection and Data Extraction

Citations from the search results were imported from the bibliographic databases into EndNote for screening for eligibility based on the eligibility criteria given above. Duplicates were removed and the remaining citations assessed by title or abstract, and a full text review then conducted. References of the remaining studies selected after the full text review were examined for potentially relevant articles based on the eligibility criteria. Analysis of the final selected studies was then conducted. This involved extracting data from the final selected studies into an Excel database, with key extraction variables including: author and date of publication, geographic setting, sample population characteristics, study objectives, NCD condition studied, intervention characteristics, outcomes measured, results of the intervention, study conclusions, study design, and quality. The data screening and extraction were conducted independently by two authors and any variances resolved between them.

### Quality assessment

A quality assessment was conducted, with the Newcastle-Ottawa Quality Assessment Scale (NOS) version for cohort studies used for the observational studies [14, 15]. This was selected as it is a convenient and widely used tool with proven validity and reliability and has been endorsed by Cochrane Reviews [15–17]. For the randomised controlled trial (RCT) study we applied the widely used and validated Cochrane Risk of Bias Tool[18].

The NOS assigns stars for methodological rigour based on three categories: study selection, comparability of study groups, and outcome assessment. Studies were initially assessed within each category using the coding manual for cohort studies provided by Wells *et al (see* [15] and http://www.ohri.ca/programs/clinical_epidemiology/nosgen.pdf), with letters and descriptions assigned describing how each study fulfilled each criterion. Stars were then assigned per the NOS assessment scale when the study achieved high quality within that category. Criteria which were not applicable to particular studies were listed as not applicable but factored into overall impressions regarding that study’s conclusions.

The Cochrane Risk of Bias Tool was developed to promote the assessment of quality of trials based on their risk of biased conclusions rather than focussing on reporting and methodological constraints [18]. The RCT was therefore evaluated as being at either high, low, or unclear risk of bias in several domains (selection, performance, detection, attrition, reporting, and other bias), and a descriptive justification of each conclusion was provided.

Neither NOS nor the Cochrane Risk of Bias Tool uses an established summary score or threshold of quality, with the quality assessment primarily used to assess strengths and weaknesses of each study rather than to rank studies or to screen them out.

### Synthesis of results

As the studies were heterogeneous in setting, intervention, and outcome, single effectiveness summary statistics across studies were not considered appropriate and were not estimated. Instead, a descriptive analysis of study results was reported.

## Results

### Study Selection

**Fig 1:**
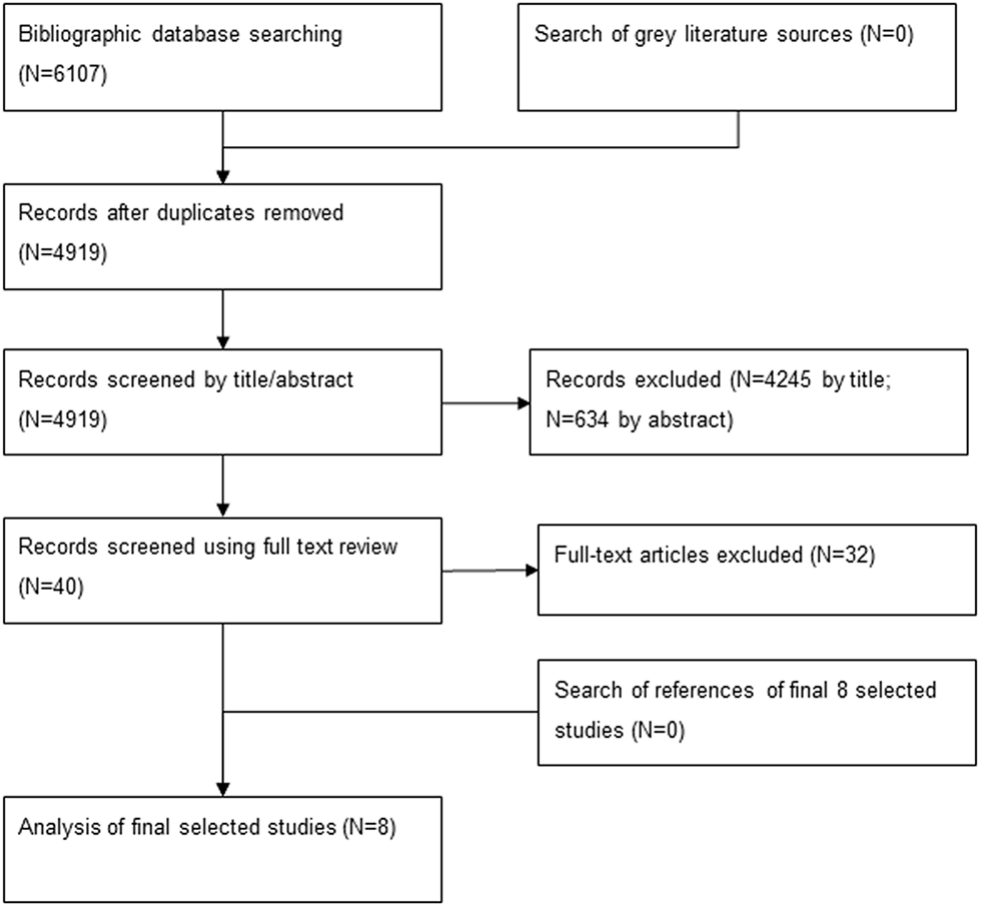
Results of screening process.

The bibliographic databases yielded 4919 citations after duplicates were removed. Of these, only 8 met the study inclusion criteria (Fig 1).[19–26] The main reasons for excluding the 4879 studies were they were: in high-income countries; not in humanitarian contexts or took place too long after a humanitarian crises; not intervention studies; did not report changes in health outcomes; or were not full papers (e.g. conference abstracts only). These reasons applied at each screening stage. Exploring references from these 8 studies did not reveal any further studies meeting eligibility criteria. No studies were identified in the grey literature.

### Study Characteristics

Key characteristics of the final 8 selected studies are included in Table 1. The studies were published between 1997 and 2014, with 6 out of 8 published within the past five years. Seven of the studies were with populations affected by armed conflict, and the remaining study with a population affected by an earthquake.[25] Sample sizes of the study populations ranged from 28 patients included in the RCT [26] to 12,550 patients in a diabetes cohort study.[24] The studies were conducted in 5 different countries: Afghanistan,[19] Georgia,[21] India,[26] Jordan,[20, 22–24] and Turkey.[25] Four of the studies[20, 22–24] came from the same research group studying Palestinian refugees in Jordan, with their studies covering diabetes and hypertension. These four studies and one other[26] took place in long-term, relatively stable refugee settings, while the remaining studies were in more acute- or early post-crisis settings.

**Table 1:** Summary of studies examining effectiveness of interventions targeting NCDs during humanitarian crises.

Author, Date [Reference]	Setting	NCD Type (study population)	Study Objectives and Design	Intervention	Outcomes Measured	Results	Study Conclusions
Bolt et al., 2010 [19]	General conflict-affected rural population in Afghanistan attending a US military hospital.	Thalassaemia (45 paediatric patients aged 13mos-11yrs)	Assess effect of palliative thalassaemia treatment in crisis setting. Case-series design.	Palliative splenectomy (programme of undeclared duration).	Change in mean Hgb/Hct; change in mean blood transfusion frequency; complications encountered.	Hgb: 5.4g/L pre-op to 8.7g/L post-op; Hct: 16.5% pre-op to 26.3% post-op; transfusion every 24 days pre-op to every~50 days post-op; complications—2 pre-op deaths, 1 post-op respiratory distress, 1 transfusion reaction, 1 case CHF post-transfusion.	Curative options likely impossible during crisis; splenectomy may be the best palliative option.
Khader et al., 2012 [20]	Camp-based Palestinian refugees in Jordan attending Nuzha primary care clinic.	Hypertension (4130 patients diagnosed with HTN).	Assess clinical outcomes of HTN care using EMR system. Assess utility of cohort monitoring using EMR in refugee context. Cohort design.	Standardised hypertension algorithm, including: diet/lifestyle management; graduated anti-hypertensive medications; referral if HTN persists; screening for HTN complications and associated conditions (e.g. DM); quarterly follow-up appointments. Cohort monitored via EMR for up to 2.5-years.	HTN clinical measures: BP, glucose, cholesterol, kidney function (creatinine) testing, medications used. Cohort monitoring: incidence/prevalence of HTN; clinic attendance (%); missed appointments; loss to f/u.	4130 patients with HTN registered in EMR (cumulative, 2.5 years): 76% remain in care; 74% of those had BP checked; 74% of those checked had BP <140/90 mmHg; 15% had 1+ complications. 226 patients assessed for 12-15-month outcomes: 62% remain in care; 76% of those meeting BP target (<140/90 mmHg); 3% glucose (DM) screened; 100% cholesterol screened; 99% creatinine screened; 8% had 1+ complications.	Mixed clinical results: approx. 3/4 of patients meeting BP targets; cholesterol, kidney function properly screened; DM poorly screened; unclear if clinical practice lacking or if data recording lacking. EMR-based cohort monitoring promising for assessing programme implementation and future needs.
Hebert et al., 2011 [21]	General conflict-affected population in Georgia (1 urban hospital and 3 rural districts).	Heart Failure (400 adult heart failure patients).	Assess clinical outcomes of a heart failure disease management programme (HFDMP). Cohort design.	2-year HFDMP: physician training; salary support; equipment supplied; patient education; free outpatient care.	Change in: ejection fraction (EF) (mean); BP (mean); BMI (mean); smoking status; health services and medication usage; NYHA HF class.	400 patients studied: 337 complete f/u, 51 lost to f/u, 12 died in war. EF increase 4.1±2.6% (p<0.001); BP—SBP decrease 30.9±20.0 mmHg (p<0.001), DBP decrease 17.8±13.0 mmHg (p<0.001); BMI statistically unchanged; smokers decrease 18.3% (p<0.001); ER use decrease 40.7% (p<0.001); hospital admission decrease 52.5% (p<0.001); beta-blocker use increase 73.3% (p<0.001); NYHA HF class—increase in Class I (+13.7%) and Class II (+19.2%), decrease in Class III (-26.0%) and Class IV (-6.8%); patients lost to f/u more likely rural.	HFDMP was able to affect clinical outcomes in a LMIC experiencing war.
Khader et al., 2012 [22]	Camp-based Palestinian refugees in Jordan attending Nuzha primary care clinic.	Diabetes Mellitus (2851 patients with DM).	Assess clinical outcomes of DM care using EMR system. Assess utility of cohort monitoring using EMR in refugee context. Cohort design.	Standardised DM algorithm, including: diet/lifestyle management; graduated anti-DM medications, including insulin if necessary; screening for DM complications and associated conditions (e.g.: HTN); quarterly follow-up appointments. Cohort monitored via EMR up to 2.5 years.	DM clinical measures: 2-hr post-prandial blood glucose; BP, cholesterol, kidney function (creatinine) testing; foot assessment; ophthalmology referral. Medications used. Cohort monitoring: incidence/prevalence of DM; clinic attendance (%); missed appointments; loss to f/u.	2851 patients with DM registered in EMR (cumulative, 2.5 years): 70% remain in care; 42% of those had 2h-PPBG checked; 50% of those checked had PPBG ≤180 mg/dl; 18% had 1+ complications. 117 patients assessed for 12-15-month outcomes: 61% remain in care; 58% of those meeting DM target (≤180 mg/dl); 100% cholesterol screened; 99% creatinine screened; 3% foot checked; no data on ophthalmology referrals; 10% had 1+ complications.	Mixed clinical results: >half of patients not receiving proper PPBG checks; half of those checked poorly-controlled; cholesterol, kidney function properly screened; DM complications poorly screened; unclear if clinical practice lacking or if data recording lacking. EMR-based cohort monitoring promising for assessing programme implementation and future needs.
Khader et al., 2014 [23]	Camp-based Palestinian refugees in Jordan attending Nuzha primary care clinic.	Diabetes Mellitus (119 patients with DM).	Assess 12-, 24-, and 36-month clinical outcomes and complications of DM care using EMR system. Assess 3-year utility of cohort monitoring using EMR in refugee context. Cohort design.	Standardised DM algorithm, including: diet/lifestyle management; graduated anti-DM medications, including insulin if necessary; screening for DM complications and associated conditions (e.g.: HTN); quarterly follow-up appointments. Cohort monitored via EMR for up to 3 years.	DM clinical measures: 2-hr post-prandial blood glucose; BP, cholesterol, kidney function (creatinine) testing; BMI; DM complications. Cohort Monitoring: baseline prevalence of DM; clinic attendance (%); missed appointments; loss to f/u.	119 patients with DM assessed at 12-, 24-, and 36-months: 72/64/61% remaining in care at 12-/24-/36-months (χ2 test-for-trend = 47.9; p<0.001); 9/19/29% lost to f/u at 12-/24-/36-months (χ2 test-for-trend = 43.5; p<0.001); 71/78/71% meeting DM goal (PPBG ≤180 mg/dl) at 12-/24-/36-months; 7/14/15% with 1+ complications at 12-/24-/36-months.	Mixed clinical results: approx. one-quarter of patients consistently missing DM goals; loss to f/u and complications rise over time; data indicate more aggressive treatment may be necessary. EMR-based cohort monitoring useful to highlight programme effects and future needs.
Khader et al., 2014 [24]	Camp-based Palestinian refugees in Jordan attending 6 primary care clinics.	Diabetes Mellitus (12550 patients with DM; focus on 288 newly registered cases).	Assess new and cumulative patient characteristics and clinical outcomes of DM care using EMR system. Assess utility of cohort monitoring using EMR in refugee context across multiple primary care clinics. Design: cohort	Standardised DM algorithm, including: diet/lifestyle management; graduated anti-DM medications, including insulin if necessary; screening for DM complications and assoc. conditions (e.g.: HTN); quarterly follow-up appointments. Cohort monitored via EMR across 6 clinics (up to 2 years at 5 clinics, 3.5 years at 1 clinic).	DM clinical measures: 2-hr post-prandial blood glucose; BP, cholesterol, kidney function (creatinine) testing; BMI; foot assessment; ophthalmology referral; DM complications and associated risk factors. Cohort monitoring: incidence/prevalence of DM; clinic attendance (%); missed appointments; loss to f/u.	12550 patients with DM registered in EMR (cumulative; 2 years at 5 clinics, 3.5 years at 1 clinic): 78% remaining in care; males more likely to be smokers (OR M:F = 7.4 (CI 6.6–8.2; p<0.001)) and inactive (OR M:F = 1.8 (CI 1.6–1.9; p<0.001)) and to have 1+ complications (OR M:F = 1.6 (CI 1.4–1.8; p<0.001)); females more likely obese (OR M:F = 0.34 (CI 0.32–0.37; p<0.001)); 99% had PPBG measured; 65% at goal (≤180 mg/dl); 99% had cholesterol measured; 63% at goal (<200 mg/dl); 99% had BP measured; 87% at goal (<140/90 mmHg); 100% had BMI measured; 40% non-obese (<30 kg/m2).	Mixed clinical results: success testing cohort widely; clinical goals not broadly met; high numbers with associated risk factors. EMR-based cohort monitoring useful to highlight programme effects and future needs.
Sever et al., 2004 [25]	General urban and rural population affected by earthquake in Marmara region of Turkey (8 HD centres).	Chronic Kidney Disease (8 HD centres responsible for 439 patients with chronic kidney disease).	Assess clinical outcomes and infrastructure changes of haemodialysis centres affected by earthquake damage. Interrupted time series design.	Haemodialysis	Clinical outputs of HD centres: total number of HD visits, % patients receiving weekly HD. Clinical outcomes: patient weight, BP. HD infrastructure: number of HD centres, machines, patients served.	8 HD centres assessed: HD machines: 95 pre-earthquake; 74 (1mo) and 79 (3mos) post-earthquake; HD personnel: 112 pre-earthquake; 86 (1mo) and 94 (3mos) post-earthquake; HD patients: 439 pre-earthquake; 175 (1wk), 239 (1mo), and 288 (3mos) post-earthquake; HD sessions: 1093/wk pre-earthquake; 520/wk (1wk), 616/wk (1mo), and 729/wk (3mos) post-earthquake; % weekly HD: 2.3% pre- to 7.2% 1wk-post-earthquake. Interdialytic weight gain: 2.9±1.1kg pre- to 2.6±1.1kg 1wk-post-earthquake; BP stable throughout.	Infrastructure damage significantly impairs HD treatment during disasters. Increase in once-weekly HD but interdialytic weight gain not increased. Patient education and disaster planning may prevent adverse outcomes.
Ryan, 1997 [26]	Tibetan refugee in non-formal refugee communities in northern India.	Arthritis (28 patients with arthritis (24 OA, 4 RA), in 14 matched pairs).	Compare limb mobility in matched pairs of Tibetan refugees with arthritis after either traditional Tibetan treatment or Western medications. RCT design.	Traditional Tibetan arthritis treatment (3 months); herbal pills; dietary restriction; behavioural advice; Western arthritis treatment (3 months); Ibuprofen or Indomethacin.	Limb mobility assessed via praxis-based scale (0–5) for active movement; pain assessed via Visual Analogue Scale.	Limb mobility: Traditional Tibetan treatment led to greater improvement in 12/14 matched pairs; 2 pairs were a draw; Mean improvement 1.39 (SD 0.59) points using traditional Tibetan treatment; 0.57 (SD 0.33) points using Western treatment.) Pain—Western treatment led to better pain improvement (data not given).	Traditional Tibetan treatment led to better arthritis improvement compared to Western treatment when assessed via limb mobility. RCTs are practicable in traditional settings.

Acronyms: BMI–body mass index; BP–blood pressure; DBP–diastolic blood pressure; DM–diabetes mellitus; EF–ejection fraction; EMR–electronic medical record; ER–emergency room; f/u–follow-up; Hct–haematocrit; HD–haemodialysis; HFDMP–heart failure disease management programme; Hgb–haemoglobin; HTN–hypertension; LMIC–low/middle-income country; mmHg–millimetres of mercury; NCD–non-communicable disease; NYHA HF class–New York Heart Association heart failure classification; OA–osteoarthritis; OR–odds ratio; PPBG–post-prandial blood glucose; RA–rheumatoid arthritis; RCT–randomised controlled trial; Ref#—reference; SBP–systolic blood pressure; SD–standard deviation.

Of the 8 studies, 7 used observational study designs[19–25] and 1 was an RCT.[26] The observational studies consisted of 5 cohort designs,[20–24, 26] 1 case series[19] and 1 interrupted time series.[25] The RCT[26] was the only study to compare outcomes between two groups. None of the studies examined the cost of implementing the intervention or the cost-effectiveness of the intervention.

The studies examined a broad range of NCD conditions: arthritis,[26] chronic kidney disease,[25] diabetes,[22–24] heart failure,[21] hypertension,[20] and thalassaemia.[19] All studies examined outcomes at the individual patient level and were primarily focused on disease management rather than prevention or health promotion. Details of each intervention and key outcome measures, study results, and specific study conclusions are presented in Table 1.

### Quality Assessment

The quality assessment identified a number of common weaknesses. The observational studies (assessed using NOS) were generally adequate in describing the study population and establishing exposure. Deficiencies common to the observational studies were predominantly related to comparability and follow-up. None had a defined comparison group or unexposed cohort. Study transparency was also noted to be a weakness common to the observational studies. No study addressed potential biases, nor did any study discuss how missing data were handled. Only three of the observational studies adequately reported follow-up periods of participants, and most studies inadequately described their follow-up procedures. Follow-up periods ranged from undefined[19] to three years[23], with studies from settings of chronic crisis demonstrating longer follow-up. Outcome assessment was also problematic. Most studies only provided self-reported outcomes; the outcomes reported by the Khader *et al*. papers were of slightly higher quality in that they were linked to electronic medical records, but those assessments were not described in a standardised way such as via the International Classification of Disease (ICD) codes. Only four studies[20–22, 24] partially discussed study limitations, and only one[23] gave any information on sources of funding, and even then only in the online version of the article.

The RCT study from India[26] was assessed using the Cochrane Risk of Bias Tool and was judged to have a high risk of selection, performance, and detection bias, primarily due to the study’s lack of blinding. The study used an open enrolment process and all members of the research team appear to have had knowledge of patients’ treatment. Although outcome reporting was a strength of the study, there was an overall high risk of additional biases given that a single non-blinded researcher assessed the outcomes. Further deficiencies surrounded the reporting of the randomisation process, which was not described in any detail. Further details on the scoring for individual studies are given in S2 File and S3 File.

### Synthesis of results

Cardiovascular diseases were assessed via two cohort studies–one in Georgia examining a heart failure disease management programme[21] and one in Jordan examining hypertension care among Palestinian refugees.[20] The contexts of these two studies differed in the sense that the study in Georgia was examining the effectiveness of a health programme that then experienced the outbreak of war during the intervention, while the study in Jordan took place in a long-term refugee setting that was relatively stable during the study period. Both studies focused on the implementation of disease management algorithms in settings of humanitarian crisis and attempted to highlight both the feasibility and challenges of such programmes.

In the study in Georgia by Hebert *et al*.,[21] the heart failure disease management programme saw some success among its 400 patients by demonstrating a statistically significant increase in ejection fraction—the fraction of blood volume exiting the heart’s ventricles with each heartbeat, which tends to decrease in the most common types of heart failure—and a statistically significant decrease in blood pressure over the course of the 2-year programme. Ejection fraction improved by 4.1±2.6% (p<0.001) and systolic and diastolic blood pressures decreased by 30.9±20.0 mmHg and 17.8±13.0 mmHg, respectively (p<0.001 for both). The intervention also demonstrated a decrease in smoking rates and in emergency room visits and hospitalisations. Heart failure classification also improved.

The study by Khader *et al*. on a hypertension management programme in Jordan had mixed clinical results, with approximately three quarters of patients meeting blood pressure targets.[20] The intervention focused on the method of cohort monitoring by using an electronic medical record system to enrol patients in a cohort that could be studied over time. The monitoring allowed researchers to also assess if goals of care were being met, both with respect to hypertension care goals such as blood pressure monitoring and with respect to associated diseases such as hypercholesterolaemia and diabetes. Results were mixed; among a sub-cohort of 226 patients assessed for 12–15 months, 100% were screened for high cholesterol but only 3% were screened for diabetes using a glucose blood test. The study authors concluded that the interventions were an improvement on baseline care in both settings. However, no comparison group was included.

Three cohort studies, all by Khader *et al*., focused on diabetes care among Palestinian refugees in long-term refugee settings in Jordan.[22–24] All three studies conducted very similar interventions consisting of a standardised diabetes protocol and assessment of patient outcomes and programme outputs via electronic medical records-based cohort monitoring. The concept was very similar as well to the aforementioned study targeting hypertension in a similar patient population with the initial DM study essentially mirroring that design.[20] The subsequent two diabetes studies differed in terms of follow-up and scope, with one study[23] focusing on 12-, 24-, and 36-month outcomes, and the other on the expansion of the programme from one clinic to six.[24] While the Khader *et al*. studies had similar designs and settings, it was confirmed via correspondence with the studies’ authors that the populations of each study differed. For this reason—and because this review featured descriptive analysis rather than meta-analysis—it was felt that inclusion of each study for analysis was appropriate.

In general, the diabetes studies claimed an improvement in the programme over time. Earlier assessment of the programme [22] found that over half of patients were not receiving post-prandial blood glucose checks and that those checked only demonstrated proper diabetes control (</ = 180mg/dl) half of the time. Subsequent assessment described in 2014[24] found that most programme outputs had improved, with nearly all patients attending clinic meeting the blood testing goals. However, other treatment goals, specifically foot examination and ophthalmology referral, that were problematic during the earlier study did not continue to be assessed in the subsequent studies. These studies also found that loss to follow-up rose over time (Γ^2^ test-for-trend = 43.5; p<0.001). Nevertheless, the study authors contend that having a monitored cohort using an electronic medical record-based system could allow for improved retention of patients through more proactive patient monitoring.

Chronic kidney disease was assessed by one retrospective study from Sever *et al*. in the Marmara region of Turkey in the aftermath of an earthquake.[25] This study focused on both the infrastructure changes and clinical patient outcomes of providing haemodialysis to patients with severe chronic kidney disease. The study found that infrastructure for providing haemodialysis was affected by the earthquake, with an acute decrease in haemodialysis centres, machines available, personnel, and subsequently numbers of haemodialysis treatments provided. Gradually these numbers improved during follow-up. The initial earthquake also led to an increase in the number of patients receiving once-weekly (i.e., less frequent) haemodialysis.

Despite the infrastructure challenges, the authors found that mean interdialytic weight gain—the amount of weight patients gain between treatments, typically fluid weight due to poor blood filtration and urine production—actually decreased from a pre-earthquake 2.9±1.1 kg to 2.6±1.1 kg 1-week post-earthquake, despite the increased numbers of patients receiving haemodialysis less frequently. Moreover, the blood pressures of patients studied remained stable throughout the study period. The authors contend that adequate patient education regarding disaster preparedness and fluid restriction likely helped mitigate poor patient outcomes, although no comparison between the baseline health status of patients able to seek care after the earthquake and the status of the larger number of patients receiving haemodialysis before the earthquake was conducted.

The only RCT eligible for inclusion in this systematic review studied changes in limb mobility among 14 matched pairs of arthritis patients living in a stable Tibetan refugee setting in northern India.[26] In this open, non-blinded RCT, patients were randomised to receive three months of either traditional Tibetan arthritis treatments (herbal pills, dietary restriction, and behavioural advice) or Western medication (ibuprofen or indomethacin). In 12 of 14 pairs, the traditional Tibetan treatment led to greater improvement in limb mobility, and in the remaining 2 pairs the treatments performed equally well. Although they have not presented the data, the authors do suggest that pain control was better with the Western treatment than the traditional treatment. The authors state that a secondary objective of the study was to examine the process of conducting an RCT on traditional treatment options, although the authors do not comment specifically on the nuances of conducting an RCT in unstable settings.

One case series study by Bolt *et al*. examined thalassaemia among 45 paediatric Afghan patients seeking care at a United States-managed military hospital in a chronic crisis setting in Afghanistan.[19] The research team provided the intervention—palliative splenectomy—with the rationale that more curative treatment (e.g. stem-cell transplantation) would not be feasible in the Afghan context. The study reported an improvement in anaemia with mean haemoglobin levels rising from 5.4g/L pre-operatively to 8.7g/L post-operatively. Furthermore, frequency of blood transfusion decreased from every 24 days to approximately every 50 days before and after surgery. The authors state that families were pleased with the improvements during follow-up, although patient-specific data, confidence intervals, and specifics regarding follow-up were not provided.

## Discussion

To the best of our knowledge, this is the first systematic review to examine the evidence of effectiveness of interventions targeting NCDs in humanitarian crises. It highlights major gaps in evidence on NCD interventions in humanitarian crises, with only eight studies meeting inclusion criteria. While the selected studies addressed a range of NCDs, there were some notable absences—particularly studies for cancer treatment and respiratory diseases. In the case of cancer, the challenges of financing and sustaining cancer care for Syrian refugees have been highlighted and further research is required on these issues.[27] In addition, none of the studies examined the effectiveness of NCD prevention activities despite prevention being central to global efforts to tackle NCDs,[9] the potential risk-factors for NCDs in crisis and fragile settings,[28] and humanitarian agencies noting the importance of NCD prevention activities.[29] Nor did any study prioritise preparedness for crises in relation to NCD management. Geographically, the studies predominantly focused on the Middle East (which is understandable given the greater burden of NCDs in the region), and studies in more resource poor settings with weaker health systems are required. There was also a high risk of bias in the identified studies.

While it is unwise to draw any definitive conclusions from such a small body of evidence, there were a number of findings that warrant further discussion. First is the apparent success of algorithm-based interventions. In Georgia, the improved clinical outcomes and use of appropriate medication showed the effectiveness of the heart failure disease management programme there.[21] Diabetes care was also implemented using an algorithm in Palestinian refugee clinics in Jordan.[22–24] Here too, the advantage of specific clinical measures led to improvement in programmatic outputs over the years of follow-up. Alongside streamlined clinical measures, it may be beneficial to include certain NCD medications on essential medication lists to facilitate their accessibility and use during a crisis. Second, the benefit of cohort monitoring using electronic medical records was highlighted.[20, 22–24] These studies were originally derived from similar cohort monitoring research conducted with other chronic diseases such as tuberculosis and HIV,[30–33] and it has been suggested links between NCDs and other chronic disease programmes such as tuberculosis and HIV could facilitate this monitoring as well as hasten implementation of NCD-focussed programmes.[34] The systematic collection of baseline and routine NCD data over time should be strongly supported, and agencies such as UNHCR have now begun implementing a standardised health information system for refugees.[35] Ideally, this monitoring should be done electronically, and given the trend toward cheaper and more mobile electronic options, the incorporation of electronic medical record technology appears to hold promise for the rapid implementation of cohort monitoring during crisis. Third, the studies also highlight the importance of capacity-building and preparation of local health staff and patients in effecting good clinical practice,[21] monitoring processes,[24] and supporting medication adherence and adaptability among patients.

In addition to the limited number of studies, the strength and quality of the existing evidence was also generally quite limited. Most of the studies used cohort study designs and while some were able to consistently follow-up over time in order to measure changes in NCD outcomes, none included a comparison group not receiving the tested intervention. This omission therefore limits conclusions on the effectiveness of the intervention. Where logistically and ethically appropriate, it would be of considerable value to include some form of comparison group in order to formulate a more robust assessment of the intervention effectiveness. The use of stepped wedge designs may be a useful approach to follow in such settings.[36] Where the use of controls is not possible, statistical methods such as interrupted time-series analysis could prove useful.[37]

Other common weaknesses include lack of discussion on how missing data were addressed, and also on other potential biases in study designs and analyses. For example, the haemodialysis study in Turkey[25] was prone to recall bias as each time point analysed was based on questionnaires sent six months after the earthquake. The RCT examining arthritis[26] was weakened in its claims by a lack of blinding. Adequate patient follow-up was another area of weakness. While loss to follow-up may be expected in the volatile and transient settings of humanitarian crisis, the lack of analysis to address it is problematic. Adjusting for potential cofounding was also not conducted (and this was further undermined by the lack of control groups).

There are also issues regarding the appropriateness and generalisability of some of the studies for other humanitarian contexts. For example, while the thalassaemia study for civilians in Afghanistan provided an intervention that was tailored toward the resources and context of Afghanistan, it nevertheless took place in a well-resourced US military hospital.[19] A number of the studies[20, 22–24, 26] were conducted in long-term refugee settlements that were relatively stable and so there is little evidence from more insecure and volatile settings.

The lack of cost considerations across all studies further limits the generalisability of the evidence. While cost-effective interventions targeting NCDs in LMICs have been developed,[9, 38] further work needs to be done to better understand the feasibility and cost of NCD interventions in humanitarian crises given their different resources and the inherent security and logistical constraints in such settings. Such information on costs and financing of NCDs is crucial to address operational and ethical issues relating to the sustainability of providing NCD care in such settings—particularly in relation to tension between commencing long-term NCD care and the shorter-term mandates of many humanitarian agencies.

Most of the evidence identified in this review is from relatively stable settings. This highlights the challenges to implementing rigorous research during an acute crisis giving the security and resource constraints and rapid population movement. However, previous longitudinal research with conflict-affected populations in volatile contexts on treatment for chronic conditions such as HIV has shown that such research is possible.[39] Given the time constraints in such settings, planning research designs in advance, pre-approving protocols, and using innovative designs that can also be rapidly implemented is recommended (e.g. using routine NCD data for cohort designs or stepped wedge designs as services are rolled out). Ethical concerns regarding intervening on conditions that require long-term care when the humanitarian response may be brief must be considered within the humanitarian and research communities, but the ethical implications of withholding an intervention or researching its effectiveness must also be strongly considered. Humanitarian donor agencies should also consider longer-term funding cycles and greater financial support for impact evaluation in order to understand the actual effectiveness of the health interventions they fund. Operational humanitarian agencies should also give greater priority to research and impact evaluation for NCDs, following the example of agencies such as Médecins Sans Frontières who have placed a relatively strong emphasis on rigorous operational research. Stronger links should also be fostered between humanitarian agencies and academia to strengthen NCD research in humanitarian contexts, and recent initiatives on this such as R2HC are to be welcomed.[40]

## Limitations

Only descriptive analysis was used, but alternative methods such as meta-analysis were not appropriate because of the multiple outcomes, interventions, study types, and the limited number of studies. The review searched only quantitative studies as the focus was on the effectiveness of interventions, and only studies from 1980 onward were included. Analysis of qualitative research examining aspects such as health care provider and user perspectives on NCD interventions in humanitarian crises would be extremely valuable. There is also the possibility that humanitarian agencies may not have published all their existing research (either as published or grey literature), and it is difficult to ascertain the potential levels of such non-publication. We could have tried hand searching humanitarian agency reports to attempt to find additional studies. However, our prior experience and discussions with humanitarian agency staff suggest it would be extremely unlikely to yield any further studies that would not have been published in scientific journals. The NOS tool used for the quality assessment in this review has been criticized for limited inter-rater reliability.[41] We did not observe any substantial discrepancies between quality assessors for this review but did not calculate inter-rater reliability.

## Conclusions

Research during humanitarian crises is inherently difficult. Researching NCDs is arguably even harder as their chronic nature tends to demand more substantial follow-up. Nevertheless, this review has highlighted an urgent need to substantially expand research on NCD interventions in humanitarian crises given their growing disease burden. Currently available studies represent an attempt to rectify this knowledge gap but are few in number and of relatively limited quality. The findings point toward the success of standardised algorithms that can be implemented consistently and monitored via patient tracking using electronic medical records. Key research needs include: a better understanding of NCD delivery models in more acute and early recovery settings; using comparison groups (where appropriate); analysing the costs and sustainability of interventions; and developing methods to minimize bias in setting where standard randomised control studies are not feasible. Such work would support the generalisability of NCD intervention findings and provide much needed guidance in this neglected field.

## Supporting Information

S1 File Table. Search terms.
(DOCX)

S2 File Table. Quality assessment of observational studies using NOS criteria.
(DOCX)

S3 File Table. Quality assessment of RCT study using Cochrane Risk of Bias Assessment Tool.
(DOCX)

S4 File Table. PRISMA Checklist.
(DOC)

S5 File Original dataset. (XLSX)
