# Supplementary material for: The Effectiveness of Interventions for Non-Communicable Diseases in Humanitarian Crises: A Systematic Review
Source: PLoS One. 2015 Sep 25;10(9):e0138303. doi: 10.1371/journal.pone.0138303 (PMC4583445; doi:10.1371/journal.pone.0138303)
Supplement: S1 File — Search terms. (DOCX) [file pone.0138303.s001.docx]

**S1 File Table. Search terms**

| **Published literature** | |
| --- | --- |
| MEDLINE/Embase/Global Health/PsychInfo Search Terms:   1. exp Disasters/ 2. exp Relief Work/ 3. Rescue Work/ 4. Emergencies/ 5. Emergency Medicine/ 6. Emergency Medical Services/ 7. Disaster Medicine/ 8. Mass Casualty Incidents/ 9. Emergency Responders/ 10. Medical Missions, Official/ 11. (humanitarian adj2 (crisis or crises or relief or response or agenc$)).tw. 12. humanitarian.tw. 13. (disaster adj3 (relief or plan$)).tw. 14. ((relief or aid) adj2 work$).tw. 15. Refugees/ 16. (refugee or evacuee or evacuated).tw. 17. (displace$ adj2 (force$ or population or human or internal$).tw. 18. Altruism/ 19. exp War/ 20. war.tw. 21. ((armed or zone) adj2 conflict$).tw. 22. (conflict affected adj3 (population$ or person$ or communit$)).tw. 23. Avalanches/ 24. Earthquakes/ 25. Floods/ 26. Landslides/ 27. Tidal Waves/ 28. Tsunamis/ 29. Cyclonic Storms/ 30. (typhoon$ or hurricane$ or cyclone$).tw. 31. (avalanche$ or earthquake$ or flood or floods or flooding or flooded or landslide$ or tsunami$).tw. 32. (disaster adj2 (natural or victim)).tw. 33. Droughts/ 34. drought$.tw. 35. Starvation/ 36. (starvation or famine$).tw. 37. 1 or 2 or 3 or 4 or 5 or 6 or 7 or 8 or 9 or 10 or 11 or 12 or 13 or 14 or 15 or 16 or 17 or 18 or 19 or 20 or 21 or 22 or 23 or 24 or 25 or 26 or 27 or 28 or 29 or 30 or 31 or 32 or 33 or 34 or 35 or 36 38. Randomized Controlled Trial/ 39. Controlled Clinical Trial/ 40. Cross-Sectional Studies/ 41. Case-Control Studies/ 42. Cohort Studies/ 43. Pilot Projects/ 44. (random$ or controlled).tw. 45. (control adj3 (area or cohort? or compare? or condition or design or group? or intervention? or participant? or study)).ab. not (controlled clinical trial or randomized controlled trial).pt. 46. ((evaluat$ or prospective or retrospective) adj1 study).tw. | MEDLINE/Embase/Global Health/PsychInfo Search Terms (cont.):   1. (quasi-experiment$ or quasiexperiment$ or quasi random$ or quasirandom$ or quasi control$ or quasicontrol$ or ((quasi$ or experimental) adj3 (method$ or study or trial or design$))).tw. 2. (time series adj2 interrupt$).tw. 3. (intervention$ or impact or effectiveness or efficacy or service$ or outcome$ or output or treatment$ or management or program$ or project$).tw. 4. Economics/ 5. Cost-Benefit Analysis/ 6. cost control.mp. or “Cost Control”/ 7. Cost savings.mp. or “Cost Savings”/ 8. cost of illness.mp. or “Cost of Illness”/ 9. cost $utility.tw. 10. (Cost$ adj2 effective$).tw. 11. cost-effective$.tw. 12. (cost adj3 utility).tw. 13. cost-utilit$.tw. 14. 38 or 39 or 40 or 41 or 42 or 43 or 44 or 45 or 46 or 47 or 48 or 49 or 50 or 51 or 52 or 53 or 54 or 55 or 56 or 57 or 58 or 59 15. Developing Countries/ 16. exp asia/ 17. exp africa/ 18. exp pacific islands/ 19. exp eastern europe/ 20. exp china/ 21. balkan peninsula/ or europe, eastern/ or transcaucasia.mp. 22. caribbean region/ or central america/ or gulf of mexico/ or latin america/ or south america.mp. 23. atlantic islands/ or indian ocean islands/ or macau/ or pacific islands/ or philippines/ or prince edward island/ or svalbard/ or west indies.mp. 24. 61 or 62 or 63 or 64 or 65 or 66 or 67 or 68 or 69 25. Japan/ 26. 70 not 71 27. 37 and 60 and 72 28. 73 29. limit 74 to yr=”1980 -Current” 30. (Non$communicable disease* or NCD* or chronic disease* or chronic condition* or long term condition* or autoimmune disease* or Lupus or heart disease or cardiovascular or cerebrovascular or stroke or hypertens* or $cholesterolaemia or heart failure or arrhythmia* or aneurysm* or cardiac or angina or myocardial infarct* or coronary heart disease or CHD or ischaem$ or cholesterol or blood pressure or blood sugar or blood glucose or diabetes or obesity or circulatory disorder* or $carditis or cardiomyopathy or anaemi* or cancer* or neoplasm* or asthma* or respiratory or COPD or chronic obstructive pulmonary disease* or pulmonary or bronchitis or lung function or lung disease* or liver function or diabetes or chronic kidney disease* or CKD or liver disease* or renal failure or cirrho* or osteoporosis or fibromyalgia or musculoskeletal or chronic pain or $arthritis or cystic fibrosis or thyroid disorder or neurological condition or Parkinson* or colitis or multiple sclerosis or MS or Alzheimer* or thrombo* or embolus or embolism or atherosclero* or vascular disease*).mp. 31. 75 and 76 |
| IBSS Search Terms:  ((humanitarian work) OR (humanitarian crises OR humanitarian hazard) OR (humanitarian aid OR humanitarian worker)) AND (Non communicable disease* OR NCD* OR chronic disease* OR chronic condition* OR long term condition* OR autoimmune disease* OR Lupus OR heart disease OR cardiovascular OR cerebrovascular OR stroke OR hypertens* OR cholesterolaemia OR heart failure OR arrhythmia* OR aneurysm* OR cardiac OR angina OR myocardial infarct* OR coronary heart disease OR CHD OR ischaemia OR cholesterol OR blood pressure OR blood sugar OR blood glucose OR diabetes OR obesity OR circulatory disorder* OR carditis OR cardiomyopathy OR anaemia* OR cancer* OR neoplasm* OR asthma* OR respiratory OR coped OR chronic obstructive pulmonary disease* OR pulmonary OR bronchitis OR lung function OR lung disease* OR liver function OR diabetes OR chronic kidney disease* OR CKD OR liver disease* OR renal failure OR cirrho* OR osteoporosis OR fibromyalgia OR musculoskeletal OR chronic pain OR arthritis OR cystic fibrosis OR thyroid disorder OR neurological condition OR Parkinson* OR colitis OR multiple sclerosis OR MS OR Alzheimer* OR thrombi* OR embolus OR embolism OR atherosclero* OR vascular disease*) | |
| **Grey literature sources** | |
| Open Grey; New York Academy of Medicine Grey Literature Report; Bielefeld Academic Search Engine (BASE); Research for Development (R4D); ReliefWeb; Desastres; Eldis; Active Learning Network for Accountability and Performance in Humanitarian Action (ALNAP); Centre for Research on the Epidemiology of Disasters (CRED); MSF Field Research; WHO Institutional Repository for Information Sharing (IRIS); UNICEF; United Nations Population Fund (UNFPA); UNHCR; International Committee of the Red Cross (ICRC); Google. | |
| Given the limited search capacity of some grey literature databases, simpler search terms such as “chronic,” “non-communicable,” and “humanitarian” were typically used. | |
