# Supplementary material for: The Effectiveness of Interventions for Non-Communicable Diseases in Humanitarian Crises: A Systematic Review
Source: PLoS One. 2015 Sep 25;10(9):e0138303. doi: 10.1371/journal.pone.0138303 (PMC4583445; doi:10.1371/journal.pone.0138303)
Supplement: S2 File — Quality assessment of observational studies using NOS criteria. (DOCX) [file pone.0138303.s002.docx]

**S2 File Table. Quality assessment of observational studies using NOS criteria**

| **Author, Date [Ref)** | **Selection** | | | | **Comparability** | **Outcome** | | |
| --- | --- | --- | --- | --- | --- | --- | --- | --- |
|  | **Representativeness of the exposed cohort** | **Selection of the non-exposed cohort** | **Ascertainment of exposure** | **Demonstration that outcome of interest was not present at start of study** | **Based on design and analysis** | **Assessment of outcome** | **Was follow-up long enough for outcomes to occur** | **Adequacy of follow up of cohorts** |
| Bolt et al., 2010 [19] | **D**  No description given | **n/a**  No comparison group | **B***  Interviews and clinical assessmen**t** | **A***  Pre- and post-surgical | **n/a**  No comparison group | **C**  Self-report | **B**  Length undefined | **D**  No statement |
| Khader et al., 2012 [20] | **A***  Long-term, urban refugees with HTN | **n/a**  No comparison group | **A***  EMR record | **B**  Not stated | **n/a**  No comparison group | **B***  Record linkage to EMR database | **A***  1 year | **D**  No statement |
| Hebert et al., 2011 [21] | **B***  Average heart failure patients | **n/a**  No comparison group | **A***  Prior clinical records and clinical screening | **A***  Outcomes focussed on changes from baseline | **n/a**  No comparison group | **C**  Self-report | **B**  Limited to 10 months; war not adequately analysed | **B***  Description given: reasons for loss to follow-up unrelated to intervention |
| Khader et al., 2012 [22] | **A***  Patients with DM who seek care | **n/a**  No comparison group | **A***  EMR record | **B**  Not stated | **n/a**  No comparison group | **B***  Record linkage to EMR database | **A***  1 year | **D**  No statement |
| Khader et al., 2014 [23] | **A***  Patients with DM who seek care | **n/a**  No comparison group | **A***  EMR record | **B**  Not stated | **n/a**  No comparison group | **B***  Record linkage to EMR database | **A***  1 year | **D**  No statement |
| Khader et al., 2014 [24] | **A***  Patients with DM who seek care | **n/a**  No comparison group | **A***  EMR record | **B**  Not stated | **n/a**  No comparison group | **B***  Record linkage to EMR database | **B**  Follow-up of new registrants undefined | **D**  Statement inadequate |
| Sever et al., 2004 [25] | **B***  Variety of HD centres representing average CKD patients | **n/a**  No comparison group | **C**  Self-report | **A***  Outcomes focussed on changes pre- and post-earthquake | **n/a**  No comparison group | **C**  Self-report | **A***  3 months follow-up adequate for acute emergency aspects | **D**  No statement |
| Quality assessment per NOS criteria for cohort studies. Stars (*) awarded if study reached threshold of high quality for that category. Letters and descriptions given to cross-reference with NOS coding manual. For further information on NOS see <http://www.ohri.ca/programs/clinical_epidemiology/oxford.asp>. For the NOS manual see <http://www.ohri.ca/programs/clinical_epidemiology/nos_manual.pdf>, and for the scoring scale see <http://www.ohri.ca/programs/clinical_epidemiology/nosgen.pdf> | | | | | | | | |
