# Supplementary material for: The Effectiveness of Interventions for Non-Communicable Diseases in Humanitarian Crises: A Systematic Review
Source: PLoS One. 2015 Sep 25;10(9):e0138303. doi: 10.1371/journal.pone.0138303 (PMC4583445; doi:10.1371/journal.pone.0138303)
Supplement: S3 File — Quality assessment of RCT study using Cochrane Risk of Bias Assessment Tool. (DOCX) [file pone.0138303.s003.docx]

**S3 File Table. Quality assessment of RCT study using Cochrane Risk of Bias Assessment Tool**

| **Cochrane Risk of Bias Assessment for Ryan, 1997 [26]** | | |
| --- | --- | --- |
| **Criterion (bias type)** | **Risk of Bias** | **Justification** |
| Random sequence generation (selection bias) | Low | Minimisation process used to generate matched pairs |
| Allocation concealment (selection bias) | High | Open enrolment and all staff aware of patients' assignments |
| Blinding of participants and researchers (performance bias) | High | Participants and researchers not blinded |
| Blinding of outcome assessment (detection bias) | High | Blinding not present; study author contends that unintended assessment bias could not produce such definitive results, but small yet consistent biases cannot be ruled out |
| Incomplete outcome data (attrition bias) | Low | One pair dropped out but unlikely to alter outcome |
| Selective reporting (reporting bias) | Low | Pre-specified outcomes have been fully reported |
| Other bias | High | Single non-blinded outcome assessor used |
